# Edge-guided feature fusion network for RGB-T salient object detection

**DOI:** 10.3389/fnbot.2024.1489658

**Published:** 2024-12-17

**Authors:** Yuanlin Chen, Zengbao Sun, Cheng Yan, Ming Zhao

**Affiliations:** Department of Information Engineering, Shanghai Maritime University, Shanghai, China

**Keywords:** saliency detection, pixel features, dynamic compensation, edge information, feature fusion

## Abstract

**Introduction:**

RGB-T Salient Object Detection (SOD) aims to accurately segment salient regions in both visible light and thermal infrared images. However, many existing methods overlook the critical complementarity between these modalities, which can enhance detection accuracy.

**Methods:**

We propose the Edge-Guided Feature Fusion Network (EGFF-Net), which consists of cross-modal feature extraction, edge-guided feature fusion, and salience map prediction. Firstly, the cross-modal feature extraction module captures and aggregates united and intersecting information in each local region of RGB and thermal images. Then, the edge-guided feature fusion module enhances the edge features of salient regions, considering that edge information is very helpful in refining significant area details. Moreover, a layer-by-layer decoding structure integrates multi-level features and generates the prediction of salience maps.

**Results:**

We conduct extensive experiments on three benchmark datasets and compare EGFF-Net with state-of-the-art methods. Our approach achieves superior performance, demonstrating the effectiveness of the proposed modules in improving both detection accuracy and boundary refinement.

**Discussion:**

The results highlight the importance of integrating cross-modal information and edge-guided fusion in RGB-T SOD. Our method outperforms existing techniques and provides a robust framework for future developments in multi-modal saliency detection.

## 1 Introduction

Salient object detection (SOD) aims to find the most noticeable area or target in an image, and has developed rapidly in recent years. In the context of the rapid development of computer technology and deep learning, SOD techniques are widely used in various fields, such as target tracking (Lee and Kim, 2018), video detection (Cong et al., 2019), image fusion (Zhang and Zhang, 2018; Cheng et al., 2018), target segmentation (Li et al., 2020, 2019), and so on.

SOD only using RGB images still suffer from performance degradation in the challenging of cluttered backgrounds, poor illumination, or transparent objects. In recent years, thermal cameras have been developed to capture infrared radiation from objects with temperatures above the zero. Thermal infrared iamges can help to detect significant objects. Even in complex conditions such as messy background of RGB image, weak light or dark, the objects in thermal infrared images will be prominent. Thus, RGB-Thermal SOD has become popular to overcome the above challenges by introducing the complementary modality information. Traditional RGB-T SOD methods mainly use low-level features and certain priors to detect targets, such as color contrast and background priors. In recent years, many excellent SOD methods have been proposed. Compared with single-channel RGB images, RGB-T images provide complementary saliency cues, which improve the performance of significance detection. For example, Niu et al. (2020) introduced a dual-stream boundary-aware network that integrates cross-modal feature sampling and multi-scale saliency aggregation, while Zhang et al. (2019) proposed an RGB-T salient object detection network based on multi-level CNN feature fusion, utilizing joint attention and information transfer units. Huang et al. (2020) designed a low-rank tensor learning model to suppress redundant information and enhance the correlation between similar image regions. Tang et al. (2019) introduced a method based on a coordinated sorting algorithm for RGB-T saliency detection, which employs a unified ranking model to describe cross-modal consistency and reliability.

Existing RGB-T salient object detection (SOD) methods face significant challenges due to the inherent differences between RGB and thermal images. While RGB images excel at capturing detailed textures under normal lighting conditions, thermal images are more effective in highlighting salient regions in low-light or cluttered environments. Despite the complementary nature of these two modalities, many existing methods fail to fully leverage this relationship. They often extract similar information from both RGB and thermal images, underutilizing the unique contributions of each modality. Furthermore, basic feature fusion strategies, such as concatenation or simple convolutional operations, fail to capture the deeper, more complex relationships between these modalities, limiting the effectiveness of the fused features and reducing the overall performance of salient object detection.

In addition to these challenges in feature fusion, many current approaches neglect the critical role of edge information in refining object boundaries. Accurate edge refinement is crucial for precise saliency map prediction, but it is often underexploited in existing methods. As a result, object boundaries tend to be poorly defined, and the suppression of background noise across the two modalities is inconsistent. This leads to irrelevant details being retained, further diminishing the accuracy of saliency detection. Without sufficient enhancement of salient regions or consistent background suppression, these methods fail to make full use of the combined strengths of RGB and thermal images.

To address these limitations, we propose an end-to-end edge-guided feature fusion network (EGFF-Net) for RGB-T salient object detection. Our approach is designed to fully exploit the complementary information between RGB and thermal images through a cross-modal feature extraction module (CMF), which aggregates both shared and distinct features from each modality. This module not only captures feature-wise information from each local region but also ensures effective fusion of modality-specific details, overcoming the shortcomings of existing simple fusion strategies. By leveraging atrous spatial pyramid pooling (ASPP) modules within the CMF, we also achieve a large receptive field and high-resolution feature maps, which further improve the fusion of complementary information.

Moreover, to enhance the precision of object boundaries, we introduce an edge-guided feature fusion module. This module incorporates multi-level features from RGB images to refine the edges of salient regions. Specifically, we extract edge features from the second layer of the RGB branch, which contains detailed texture information, and combine them in a cascade with features from deeper layers to guide the fusion process. This edge-guided refinement ensures that the boundaries of salient objects are more accurately captured, addressing the limitations of previous methods that overlook edge information. Additionally, our approach enhances the overall saliency representation, resulting in more effective suppression of irrelevant background details and more accurate detection of salient regions under various conditions.

By integrating multi-level edge features and applying a layer-by-layer decoding structure, our method ensures both effective feature fusion and precise saliency map prediction. The edge-guided fusion strategy, in combination with the CMF module, directly addresses the challenges of incomplete feature fusion and poor edge refinement, ultimately leading to improved performance in RGB-T SOD tasks. The main contributions of this work are as follows:

1. We proposed the structure of double-branch edge feature nested network for RGB-T SOD, which consists cross-modal feature extraction with the encoding format, salience map prediction and a layer-by-layer decoding format for the prediction of saliency targets.
2. We proposed the cross-modal feature extraction module (CMF) to extract and aggregate united features and intersecting information between two modalities. Two atrous spatial pyramid pooling modules (ASPPs) (Chen et al., 2017) are embedded into CMF module for obtaining large receptive field as well as high resolutions. Three branches, i.e., RGB-T branch, T-RGB branch, and mixed branch are designed when aggregating the transmembrane state features, so as to better retain the effective information of different modes and realize the mutual compensation between the two modalities.
3. We proposed an edge-guided feature fusion module that enables the refinement of salient target boundaries by edge information, as well as enhancing the overall salient target region and suppressing redundant information. The secondary feature extraction for edge features is designed on the cascaded feature map using a specific convolution block to obtain edge feature maps of the RGB images as the mult-level refinements of salient target boundaries.

## 2 Related works

### 2.1 RGB-T salient object detection

RGB-T Salient Object Detection (SOD) has attracted increasing attention due to the complementary nature of visible light and thermal infrared images. Tu et al. (2020) introduced a collaborative graph learning algorithm for RGB-T saliency detection, along with the VT1000 dataset containing 1000 RGB-T image pairs. Li et al. (2021) proposed a Hierarchical Alternate Interactions Network (HAINet) for RGB-D SOD, which could be adapted for RGB-T tasks by focusing on cross-modal interaction. Tu et al. (2021) proposed a dual-decoder framework that models interactions across multi-level features, modalities, and global contexts to exploit modality complementarity. However, its reliance on pre-defined interaction mechanisms limits its adaptability to dynamically changing conditions and subtle modality variations. Liu et al. (2022) introduced SwinNet, which employs Swin Transformers for hierarchical feature fusion and attention mechanisms to bridge the gap between modalities. To address image misalignment, Tu et al. (2022) presented DCNet, incorporating spatial and feature-wise transformations for modality alignment and a bi-directional decoder for hierarchical feature enhancement. While promising, DCNet's complex alignment strategy and decoding structure hinder its efficiency in real-time applications. Pang et al. (2023) introduced CAVER, a view-mixed transformer emphasizing global information alignment, and Zhou et al. (2023) presented WaveNet, a wavelet-based MLP employing a transformer teacher for cross-modality feature fusion. These methods demonstrated the potential of global alignment mechanisms but often struggled with fine-grained local details. More recently, He and Shi (2024) proposed a SAM-based RGB-T SOD framework incorporating modules such as High-Resolution Transformer Pixel Extraction to refine detection. However, its reliance on pre-trained models and complex feature extraction pipelines may limit adaptability in diverse scenarios.

Despite these advancements, RGB-T SOD remains challenging due to the inherent differences in RGB and thermal modalities, particularly in suppressing complex backgrounds and enhancing salient object boundaries. While Tu et al. (2021) proposed a dual-decoder framework to exploit modality complementarity, its reliance on pre-defined interaction mechanisms limits its adaptability to dynamic conditions. Liu et al. (2022) introduced SwinNet, which employs Swin Transformers for hierarchical feature fusion and attention mechanisms to bridge the gap between modalities. While its transformer-based architecture effectively captures long-range dependencies, its fusion strategy relies heavily on spatial alignment and channel re-calibration, which may fail to fully exploit complementary information from RGB and thermal modalities. Methods like CAVER (Pang et al., 2023), which emphasize global feature alignment, struggle with capturing fine-grained local details, particularly in the presence of background clutter. Similarly, WaveNet (Zhou et al., 2023) utilizes wavelet-based MLPs for cross-modality feature fusion, but its reliance on wavelet transforms for global feature aggregation may limit its ability to refine object boundaries and capture local texture details effectively. Tu et al. (2022) proposed DCNet to address image misalignment, but its complex alignment strategy and decoding structure hinder efficiency in real-time applications.

EGFF-Net overcomes these limitations through two key innovations: the Cross-Modal Feature (CMF) module and the Edge Embedding Decoder (EED) module. The CMF module improves modality fusion by dynamically aggregating complementary features from both RGB and thermal images, using multiple branches (RGB-T, T-RGB, and mixed) to ensure better retention of cross-modal information and mutual compensation between the modalities. The EED module, on the other hand, enhances boundary refinement by incorporating multi-level edge features from the RGB modality, which directly improves the accuracy of object boundaries, especially in cluttered environments or when thermal signals are weak. Together, these modules address the challenges of incomplete feature fusion and insufficient boundary refinement, ensuring robust saliency detection even in scenarios with complex backgrounds or misaligned images.

## 3 Methodology

**Figure 1:**
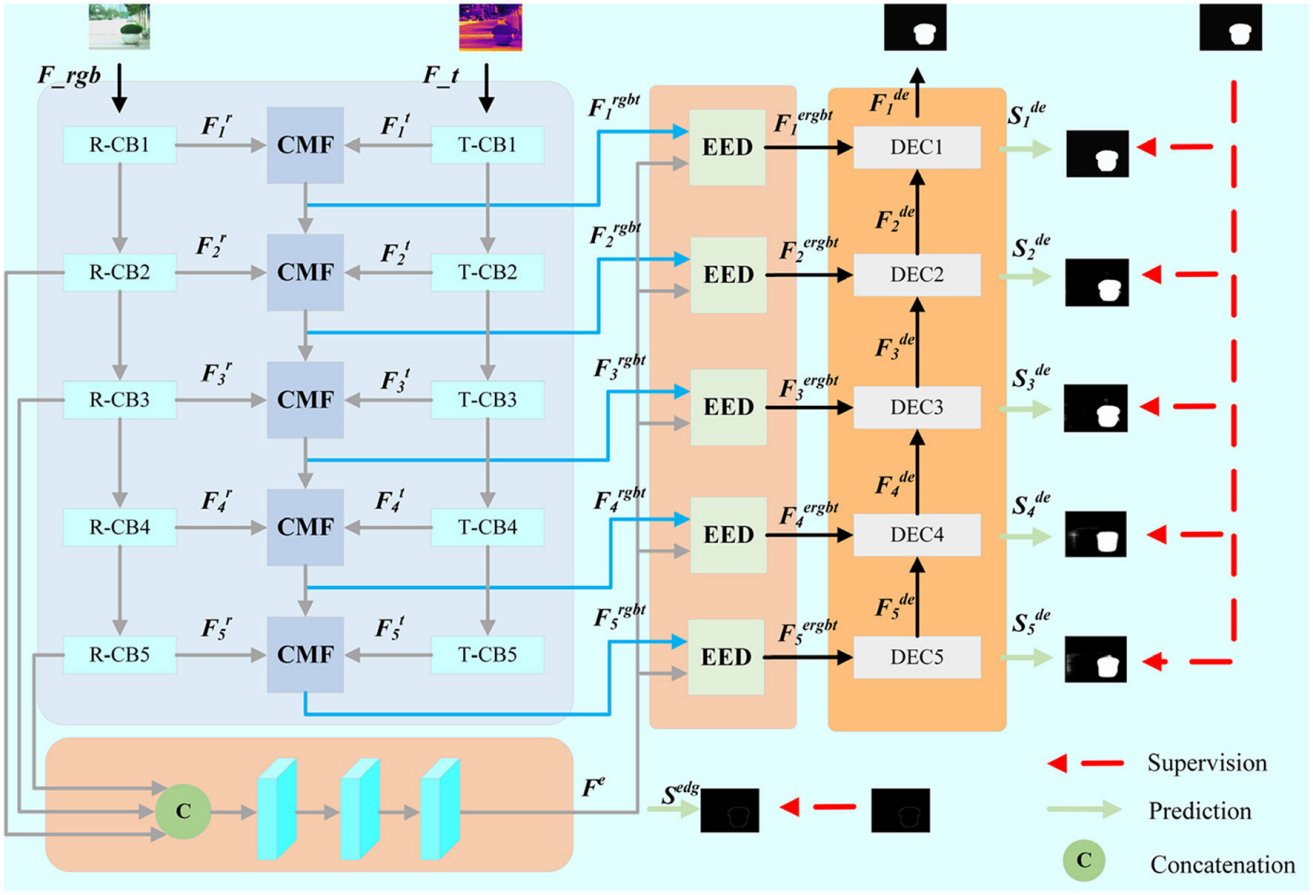
The framework of the proposed EGFF-Net. CMF and EED stand for Cross Model Fusion Module (CMF) and Edge Embedding Module (EED), respectively.

The proposed network mainly includes cross-modal feature extraction, edge-guided feature fusion, salience map prediction and hybird loss function. As shown in Figure 1, our network is a double-input end-to-end network structure, which is fed with RGB-T images. In order to better extract the features of different modes and realize the information complementarity between the feature maps of the transmembrane state, we propose the cross-modal feature extraction method, which can effectively integrate information between different modes. Considering that edge information is very helpful in refining the edge details of significant areas, edge-guided feature fusion module is explored to enhance the edge features of salient region. The cascaded decoders are used to integrate the multi-level features to generate prediction map.

### 3.1 Cross-modal feature extraction

The proposed network is based on the encoding-decoding architecture. The double-branch VGG-16 is adopted as the backbone, which only retains the previous convolution layer, and removes the last pooling layer and all full connection layers. As shown in Figure 1, the RGB-T images are fed into two branches for feature extraction. R-CB_*i*_ and T-CB_*i*_ (*i*∈{1, 2, 3, 4, 5}) are used to extract RGB and thermal features, respectively. The dual encoder outputs the i-th layer features of the RGB and thermal encoder, which denotes as ${\text{F}}_{i}^{r}$ and ${\text{F}}_{i}^{t}$ (*i*∈{1, 2, 3, 4, 5}), respectively.

**Figure 2:**
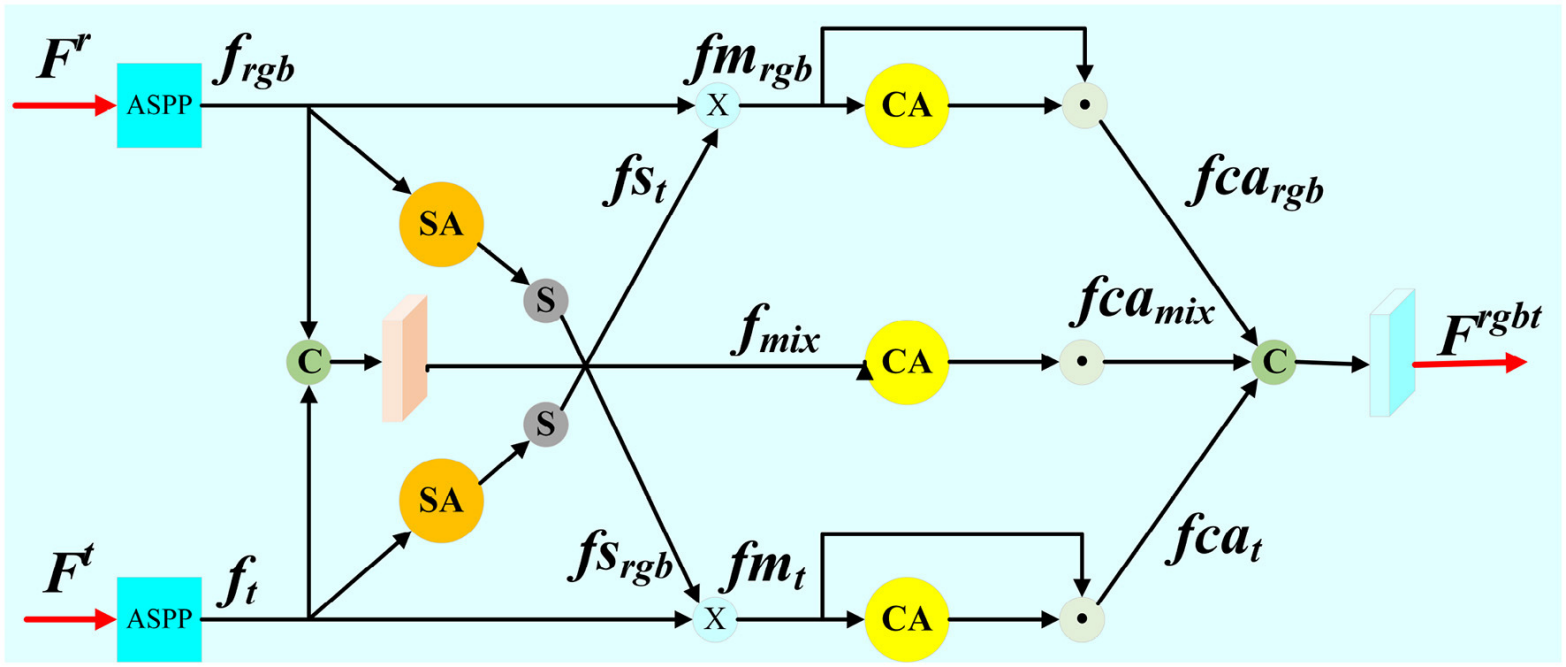
Detail illustration of proposed Cross Model Fusion Module (CMF).

In order to better fuse the information of different modes, we propose the cross model fusion module (CMF) as shown in Figure 2. Firstly, ASPPs are embedded into CMF module for obtaining large receptive field and high resolution at the same time. *f*_*rgb*_ and *f*_*t*_ are feature maps of ASPPs performing, respectively. Three branches, i.e., RGB-T branch, T-RGB branch, and mixed branch are designed in CMF module when aggregating the transmembrane state features, so as to better retain the effective information of different modes and realize the mutual compensation between the two modes.

We perform *f*_*t*_ through a spatial attention mechanism (SA), and then through the Sigmoid function (S) in RGB-T branch. The spatial proportion of the thermal feature map *fs*_*t*_ is obtained, and the pixel values range from 0 to 1. The original feature map *f*_*rgb*_ multiplies with the attention feature maps *fs*_*t*_ to obtain the auxiliary feature map *fm*_*rgb*_, which enhances the salient region of the RGB feature maps. This operation can be formally represented as:


(1) $$\begin{array}{l} \text{SA}\left(f\right)=\sigma \left(\text{conv}\left(\text{cat}\left({\text{GMP}}_{s}\left(f\right),{\text{AVG}}_{s}\left(f\right)\right)\right)\right) \end{array}$$

(2) $$\begin{array}{l} {fs}_{t}=\sigma \left(\text{SA}\left(f_{t}\right)\right) \end{array}$$

(3) $$\begin{array}{l} {fm}_{rgb}=f_{rgb}⊗{fs}_{t} \end{array}$$

where GMP_*s*_(·) represents the maximum pooling of spatial attention mechanisms, AVG_*s*_(·) represents the average pooling of spatial attention mechanisms, *conv*(·) for convolutional operation, σ(·) for Sigmoid function, *SA*(·) represents spatial attention mechanisms, ⊗ respresents matrix multiplication. T-RGB branch has the symmetrical structure with RGB-T branch. The thermal feature maps *f*_*t*_ is multiplied by the RGB feature maps *fs*_*rgb*_, which has been performed through the attention mechanism and Sigmoid function. The auxiliary feature map *fm*_*t*_ is obtained as follows:


(4) $$\begin{array}{l} {fs}_{rgb}=\sigma \left(\text{SA}\left(f_{rgb}\right)\right) \end{array}$$

(5) $$\begin{array}{l} {fm}_{t}=f_{t}⊗{fs}_{rgb} \end{array}$$

In the mix branch, RGB feature maps *f*_*rgb*_ and thermal infrared feature maps *f*_*t*_ are concatenated to extract the mixed feature *f*_*mix*_ of transmembrane states as follows:


(6) $$\begin{array}{l} f_{mix}=\text{conv}\left(\text{cat}\left(f_{rgb},f_{t}\right)\right) \end{array}$$

In the RGB-T branch, thermal features (*f*_*t*_) are processed through a spatial attention (SA) mechanism, which emphasizes salient regions by generating spatial attention weights (*fs*_*t*_) that highlight significant areas in the thermal feature map. These attention weights are then applied to the RGB feature map (*f*_*rgb*_) to produce an enhanced RGB feature map (*fm*_*rgb*_) where regions aligned with thermal saliency are emphasized. This selective enhancement effectively suppresses irrelevant details in RGB features that are not supported by thermal information. The RGB-T branch thus leverages thermal cues to refine RGB feature maps, enhancing their focus on salient regions.

The T-RGB branch complements the RGB-T branch by following a symmetric design. Here, RGB features (*f*_*rgb*_) are processed through the same spatial attention mechanism, producing spatial attention weights (*fs*_*rgb*_) that highlight significant areas. These weights are then applied to the thermal feature map (*f*_*t*_), producing an enhanced thermal feature map (*fm*_*t*_). This operation ensures that salient regions in thermal images are reinforced using RGB information, particularly in scenarios where thermal signals are weak or ambiguous. The T-RGB branch thus strengthens thermal features by leveraging complementary cues from RGB images.

While the RGB-T and T-RGB branches focus on modality-specific refinement, the mixed branch addresses cross-modal feature integration at a more global level. In this branch, RGB (*f*_*rgb*_) and thermal (*f*_*t*_) features are concatenated to form a unified feature representation (*f*_*mix*_). This concatenated feature map is processed through convolutional layers to learn mixed modality representations that capture high-level interactions between RGB and thermal modalities. The mixed branch ensures that the complementary information across both modalities is preserved and exploited to its full potential.

The channel attention mechanism is applied to obtain the weights of each channel from feature maps, and then these weights are multiplied with the corresponding feature maps. Finally, the feature maps of the three branches are concatenated, and the aggregated feature ${\text{F}}_{i}^{rgbt}$ (*i*∈{1, 2, 3, 4, 5}) is extracted as follows:


(7) $$\begin{array}{l} \text{CA}\left(f\right)=\sigma \left({\text{FC}}_{\beta }\left(\text{FC}\alpha \left({\text{GMP}}_{c}\left(f\right)\right)\right)⊕{\text{FC}}_{\beta }\left({\text{FC}}_{\alpha }\left({\text{AVG}}_{c}\left(f\right)\right)\right)\right) \end{array}$$

(8) $$\begin{array}{l} fca_{rgb}=\text{CA}\left(fm_{rgb}\right)⊙fm_{rgb} \end{array}$$

(9) $$\begin{array}{l} fca_{t}=\text{CA}\left(fm_{t}\right)⊙fm_{t} \end{array}$$

(10) $$\begin{array}{l} fca_{mix}=\text{CA}\left(f_{mix}\right)⊙f_{mix} \end{array}$$

(11) $$\begin{array}{l} {\text{F}}^{rgbt}=\text{conv}\left(\text{cat}\left(fca_{rgb},fca_{t},fca_{mix}\right)\right) \end{array}$$

where GMP_*c*_(·) represents maximum pooling of channel attention mechanisms, AVG_*c*_(·) represents the average pooling of channel attention mechanisms, FC_α_(·) represents the fully connected layer of the Relu activation function, FC_β_(·) represents the fully connected layer of the Sigmoid activation function, *CA*(·) represents channel attention mechanisms, *fca*_*rgb*_, *fca*_*t*_, *fca*_*mix*_ represent the performing of *fm*_*rgb*_, *fm*_*t*_, *f*_*mix*_ after the channel attention, respectively, ⊙ represents elemental multiplication, ⊕ represents elemental summation.

### 3.2 Edge-guided feature fusion

Considering that edge information is very helpful in refining the edge details of significant areas, edge-guided feature fusion module is explored to enhance the edge features of salient region. For the reasons that RGB images contain more detailed textures, we choose the second layer feature maps of RGB combined with the fourth and fifth layer feature maps in a cascade way to extract edge information as a guide for feature fusion. The secondary feature extraction as shown in Figure 1 is performed on the cascaded feature map using a specific convolution block to obtain edge feature maps **F**^*e*^ of the RGB images. The representation can be expressed as:


(12) $$\begin{array}{l} {\text{F}}^{e}={\text{conv}}_{edg}\left(\text{cat}\left({\text{F}}_{2}^{r},up\left({\text{F}}_{3}^{r}\right),up\left({\text{F}}_{5}^{r}\right)\right)\right) \end{array}$$

where ${\text{F}}_{i}^{r}$ represents the feature maps of the *i*-th layer, *up*(·) represents upsampling, and conv_*edg*_(·) represents edge feature extraction convolution.

**Figure 3:**
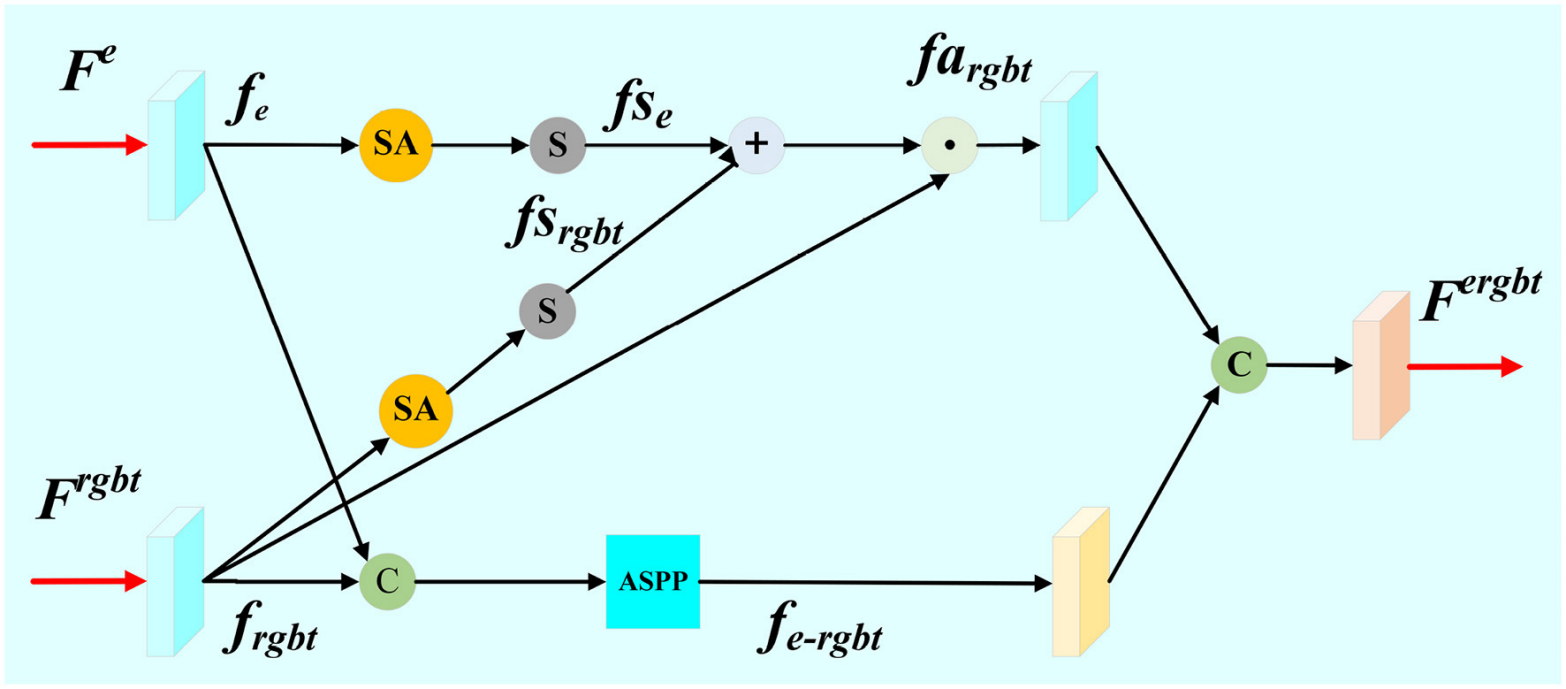
Detail illustration of proposed Edge Embedding module (EED).

Edge embedding module (EED) is designed to embed the edge feature **F**^*e*^ with the aggregated feature **F**^*rgbt*^, as shown in Figure 3. The aggregated feature **F**^*rgbt*^ and edge feature **F**^*e*^ are each subjected to one feature extraction via a convolution layer. Then, we cascade the salient region feature maps *f*_*rgbt*_ with the edge feature maps *f*_*e*_. In order to better capture the contextual information and obtain a larger perceptual field, ASPP module is employed to extract features from the cascaded feature maps. The feature map *f*_*e*−*rgbt*_ is obtained as the edge-to-significance region guide. These operations are expressed as the following:


(13) $$\begin{array}{l} f_{rgbt}=\text{conv}\left({\text{F}}^{rgbt}\right),f_{e}=\text{conv}\left({\text{F}}^{e}\right) \end{array}$$

(14) $$\begin{array}{l} f_{e-rgbt}=\text{ASPP}\left(\text{cat}\left(f_{rgbt},f_{e}\right)\right) \end{array}$$

Theoretically, the feature maps of salient region focus on the salient target, while the feature maps of edges pay more attentions on the texture of edges. In order to enhance the features of salient targets, both of the salient region feature map *f*_*rgbt*_ and the edge feature map *f*_*e*_ are performed through a channel spatial attention mechanism and Sigmoid function, respectively.


(15) $$\begin{array}{l} fs_{rgbt}=\sigma \left(\text{SA}\left(f_{rgbt}\right)\right),fs_{rgbt}\in \;\left[0,1\right] \end{array}$$

(16) $$\begin{array}{l} fs_{e}=\sigma \left(\text{SA}\left(f_{e}\right)\right),fs_{e}\in \;\left[0,1\right] \end{array}$$

Then, we perform an addition operation on *fs*_*rgbt*_ and *fs*_*e*_, and multiply the result with the map of salient region features to strengthen the saliency region as well as reduce the background interference. The feature map of the enhanced salient region *fa*_*rgbt*_ is obtained as follows:


(17) $$\begin{array}{l} fa_{rgbt}=f_{rbgt}⊙\left(\left(fs_{rgbt}+fs_{e}\right)/2\right) \end{array}$$

Finally, the two branch feature maps *fa*_*rgbt*_ and *f*_*e*−*rgbt*_ are concatenated, and be further put through a convolution block, respectively. The final edge-guided feature map **F**^*ergbt*^ is formulated as follows:


(18) $$\begin{array}{l} {\text{F}}^{ergbt}=\text{conv}\left(\text{cat}\left(\text{conv}\left(fa_{rgbt}\right),\text{conv}\left(f_{e-rgbt}\right)\right)\right) \end{array}$$

### 3.3 Salience map prediction and hybird loss function

As shown in Figure 1, the layer-by-layer decoding structure is designed for the prediction of salient maps. We cascade the output ${\text{F}}_{i}^{de}$ (*i*∈{1, 2, 3, 4, 5}) of each decoding block with the output ${\text{F}}_{i}^{ergbt}$ (*i*∈{1, 2, 3, 4, 5}) of the edge-guided feature fusion module of the upper layer, and then fed them into the upper decoding block as follows:


(19) $$\begin{array}{l} {\text{F}}_{5}^{de}={\text{conv}}_{de}\left({\text{F}}_{5}^{ergbt}\right) \end{array}$$

(20) $$\begin{array}{l} {\text{F}}_{i}^{de}={\text{conv}}_{de}\left(\text{cat}\left({\text{F}}_{i+1}^{de},{\text{F}}_{i}^{ergbt}\right)\right),i\in \left\{1,2,3,4\right\} \end{array}$$

where conv_*de*_(·) represents the decoding block. The decoding block consists of three normal convolution blocks as well as a deconvolution block, which ensures the feature maps fed into the decoding block with the same dimensions.

The total loss $L_{total}$ consists of significance loss $L_{de}$ and the edge loss $L_{edg}$. Both of them includes hybird BCE loss and IOU loss as follows:


(21) $$\begin{array}{l} L_{total}=\overset{5}{\underset{i=1}{\sum }}\left(L_{de\left(i\right)}\right)+L_{edg} \end{array}$$

(22) $$\begin{array}{l} L_{de}=L_{BCE}+L_{IOU} \end{array}$$

(23) $$\begin{array}{l} L_{edg}=L_{BCE}+L_{IOU} \end{array}$$

(24) $$\begin{array}{l} {ℒ}_{BCE}=\frac{1}{H\times W}\sum_{x=1}^{H}\sum_{y=1}^{W}[G_{xy}\log\left(up\left(S_{xy}\right)\right)+\\ \;\left(1-G_{xy}\right)\log\left(1-up\left(S_{xy}\right)\right)] \end{array}$$

(25) $$\begin{array}{l} L_{IOU}=1-\frac{\sum_{x=1}^{H}\sum_{y=1}^{W}up\left(S_{xy}\right)G_{xy}}{\sum_{x=1}^{H}\sum_{y=1}^{W}up\left(S_{xy}\right)+G_{xy}-up\left(S_{xy}\right)G_{xy}} \end{array}$$

where H and W denote the height and width of the original image, respectively. *G*_*xy*_ represents the pixel value of the ground-truth, and *S*_*xy*_ represents the probability value of the predicted regions. *up*(·) represents the bilinear upsampling.

## 4 Experiments

### 4.1 Implementation details

For a fairer comparison, we use the same datasets, the same hardware equipment and parameters with seven state-of-the-art methods, including MIDDNet (Tu et al., 2021), HAINet (Li et al., 2021), SwinNet (Liu et al., 2022), DCLNet (Tu et al., 2022), GCLNet (Tu et al., 2020), CAVERNet (Pang et al., 2023), WaveNet (Zhou et al., 2023). We conduct all experiments using the PyTorch framework, and the experiments were performed on a device equipped with an NVIDIA GeForce RTX 2080Ti GPU and 16-GB RAM. All images are resized to 224 × 224 pixels. We use an adaptive momentum estimation algorithm (ADAM) optimizer to optimize our model with a batch size of 2 for 150 epochs to train the network. The initial learning rate is set as 1 × 10^−3^, and is reduced by a tenth every 30 epoches.

### 4.2 Datasets and evaluation measures

To better evaluate the proposed scheme, we conducted RGB-T significant target detection experiments on VT5000, VT1000, VT821 datasets, respectively. VT5000 consists of 5,000 images. Two thousand and five hundred images are selected as the training set, and the other 2,500 images as well as VT1000 and VT821 as the test set for the experiments. Three evaluation metrics are used to evaluate the results of all experiments, including precision-recall (PR) curve, max F-measure(*F*_β_) (Achanta et al., 2009), mean absolute error (MAE) (Perazzi et al., 2012).

### 4.3 Results and analysis

**Figure 4:**
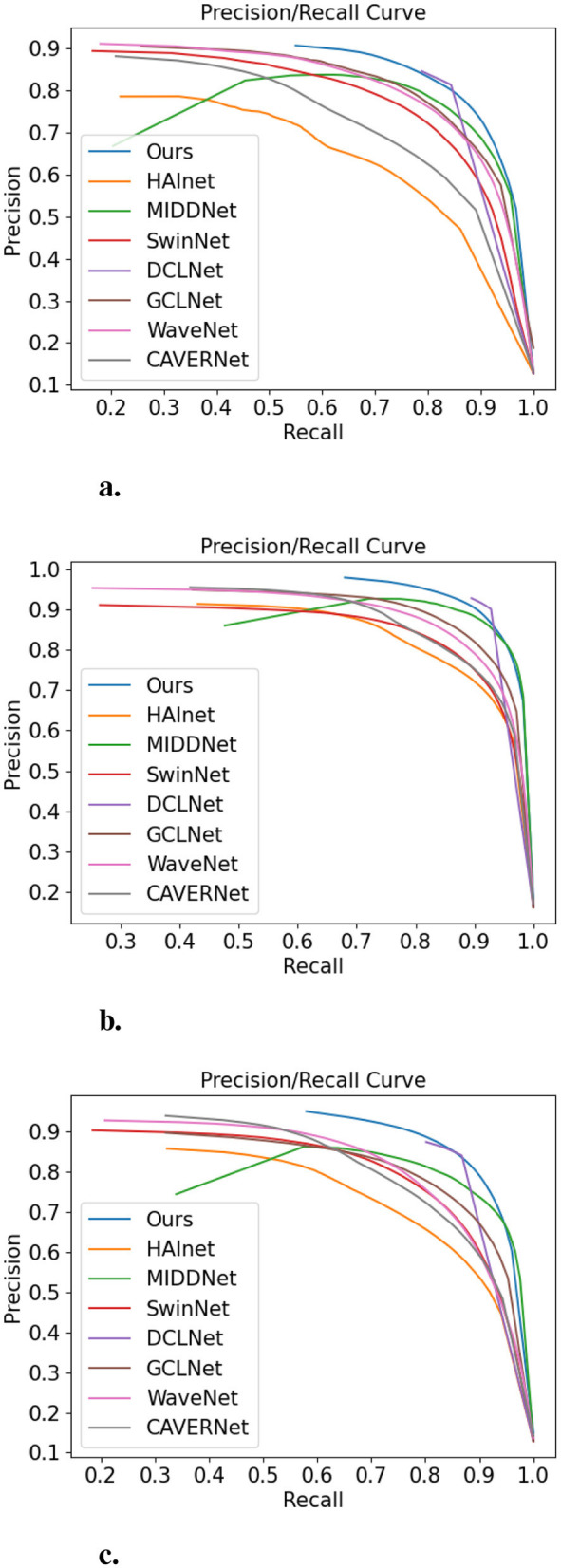
PR curves of different saliency detectors on **(A)** VT5000, **(B)** VT1000, and **(C)** VT821 datasets.

*Quantitative evaluation:* In order to present our experimental results more intuitively, we performed a quantitative analysis of the experimental data. As shown in Figure 4, the precision-recall curves provide a more intuitive and comprehensive comparison with other methods. It can be seen that the results of our proposed method P-R curve are better than several other methods. The max F-measure and mean absolute error are depicted in Table 1. The MAE value of the proposed method is smaller than the other seven models, except on the VT821 dataset, where it is slightly higher than GCLNet. As observed in Table 1, although our method outperforms state-of-the-art (SOTA) models on the VT5000 and VT1000 datasets, its performance on VT821 is slightly lower compared to GCLNet. We attribute this to GCLNet's collaborative graph learning algorithm, which leverages hierarchical deep features and superpixel-based graph nodes to jointly learn graph affinity and node saliency. This approach suits the VT821 dataset, which comprises more complex scenes with finer local structures. In contrast, our model focuses more on multimodal fusion and edge refinement on a broader scale, making it more effective on larger datasets like VT5000 and VT1000.

**Table 1:** Quantitative comparison of different models for RGB-T on three datasets.

**Methods**	**VT5000**		**VT1000**		**VT821**	
	**MAE**	**max** *F*_β_	**MAE**	**max** *F*_β_	**MAE**	**max** *F*_β_
Ours	0.0590	0.8973	0.0325	0.9024	0.0427	0.8645
MIDDNet	0.0658	0.8599	0.0381	0.8952	0.0686	0.8237
HAINet	0.0871	0.7017	0.0509	0.8291	0.0668	0.7896
SwinNet	0.0795	0.8006	0.0656	0.8381	0.0664	0.8152
DCLNet	0.0556	0.8637	0.0329	0.9017	0.0564	0.8008
GCLNet	0.0786	0.7786	0.0437	0.8577	0.0345	0.8670
WaveNet	0.0662	0.8319	0.0491	0.8749	0.0566	0.8504
CAVERNet	0.0781	0.7847	0.0510	0.8672	0.0671	0.8351

**Figure 5:**
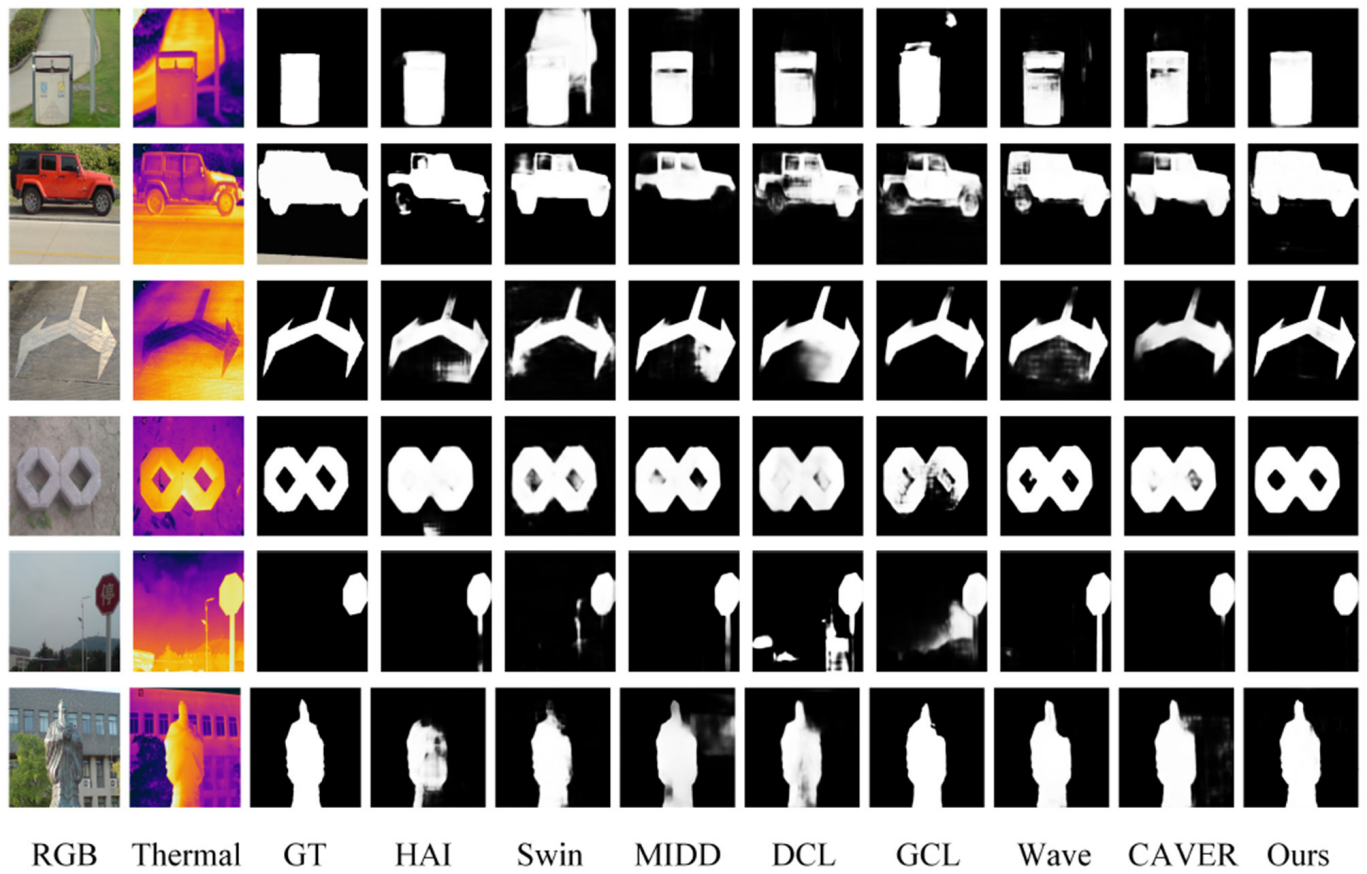
Visual comparisons of the proposed EGFF-Net and other state-of-the-art methods.

*Qualitative evaluation:* We visualize the saliency maps of the proposed methods and other seven typical models to qualitatively evaluate the performance in three challenging situations, including normal images, complex background images, and dim background images. As shown in Figure 5, the proposed method was able to capture salient areas accurately in these challenging situations.

From the quantitative and qualitative results, we conclude that the proposed method achieves excellent SOD performance. Although our method's MAE on the VT821 dataset is slightly higher than that of GCLNet, it achieves the best performance across all other metrics and datasets, including VT5000 and VT1000, where it surpasses the state-of-the-art models.

### 4.4 Ablation study

We conducted ablation experiments to verify the necessity of each component of the proposed EGFF-Net. The experiments were performed on three datasets to analyze the contribution of different modules. All the models were experimented again with the same training and test sets, and the results obtained are presented in Table 2.

- **Backbone** **+** **CMF_Sub1**: Concatenates the features from both RGB and thermal images in the CMF submodule, studying the effect of feature concatenation without the compensatory mechanism.
- **Backbone** **+** **CMF_Sub2**: Integrates the CMF submodule where the thermal infrared image compensates for the RGB image, focusing on how thermal information enhances RGB saliency while suppressing background information.
- **Backbone** **+** **CMF_Sub3**: Integrates the CMF submodule where the RGB image compensates for the thermal infrared image, aiming to explore how RGB information enhances thermal infrared saliency while suppressing background information.
- **Backbone** **+** **CMF - ASPP**: Removes the Atrous Spatial Pyramid Pooling (ASPP) module from the CMF to analyze its role in enhancing multi-scale feature extraction at the input stage.
- **Backbone** **+** **CMF**: Uses the complete CMF module, combining the compensatory mechanisms between RGB and thermal images, representing the full cross-modal fusion model.
- **Backbone** **+** **CMF** **+** **EED_Sub1**: Retains the compensatory effect of the fused features on the edge features, exploring how the enhanced feature map helps refine the edges in salient regions.
- **Backbone** **+** **CMF** **+** **EED_Sub2**: Retains the edge features' compensatory effect on the fused features, studying how edge information influences the overall saliency feature map.
- **Backbone** **+** **CMF** **+** **EED**: Implements the complete EGFF-Net model, combining both the full CMF module and edge-guided feature fusion (EED) module, representing the final architecture used for RGB-T SOD.

**Table 2:** Results of ablation studies.

**Methods**	**VT5000**		**VT1000**		**VT821**	
	**MAE**	**max** *F*_β_	**MAE**	**max** *F*_β_	**MAE**	**max** *F*_β_
Backbone + CMF_Sub1	0.0824	0.7943	0.0631	0.8563	0.0753	0.8329
Backbone + CMF_Sub2	0.0810	0.7978	0.0623	0.8567	0.0748	0.8333
Backbone + CMF_Sub3	0.0812	0.7980	0.0623	0.8571	0.0745	0.8331
Backbone + CMF - ASPP	0.0699	0.8559	0.0448	0.8787	0.0488	0.8488
Backbone + CMF	0.0681	0.8621	0.0436	0.8802	0.0502	0.8512
Backbone + CMF + EED_Sub1	0.0662	0.8803	0.0421	0.8883	0.0493	0.8588
Backbone + CMF + EED_Sub2	0.0665	0.8807	0.0423	0.8897	0.0490	0.8585
Backbone + CMF + EED	0.0590	0.8973	0.0375	0.9024	0.0427	0.8645

The results demonstrate that the proposed EGFF-Net with all modules intact consistently outperforms its variants across all datasets. Specifically, the complete EGFF-Net shows superior performance in terms of both accuracy and precision, achieving the lowest MAE across the majority of benchmarks. For instance, removing the ASPP module resulted in noticeable degradation in multi-scale feature extraction, leading to weaker performance on complex scenes with varying object sizes. This highlights the crucial role of ASPP in capturing diverse spatial features across different scales, which is particularly beneficial for RGB-T data where salient objects vary significantly in size and structure.

Further analysis reveals the indispensable contributions of the CMF module, especially its three sub-branches (CMF_Sub1, CMF_Sub2, and CMF_Sub3). CMF_Sub1, while providing a baseline fusion by simply concatenating RGB and thermal features, lacks the compensatory mechanisms necessary for addressing modality-specific challenges, limiting its ability to suppress irrelevant background information. In contrast, CMF_Sub2 effectively enhances saliency by utilizing thermal information to compensate for RGB deficiencies, particularly in low-light or high-noise conditions. Similarly, CMF_Sub3 improves saliency detection by refining thermal features through RGB guidance, which is advantageous for objects with detailed textures and boundaries. When integrated into the complete CMF module, these branches collaboratively balance global and modality-specific information, leading to significant improvements in both saliency prediction and noise suppression.

The EED module further augments these gains by addressing the limitations in boundary refinement. The submodules within EED, focusing on the mutual enhancement between edge and fused features, play a vital role in resolving challenges associated with complex object contours. The edge-to-feature compensation ensures that the global saliency map incorporates precise boundary details, while the feature-to-edge compensation reinforces edge clarity using high-level saliency cues. Without EED, the model struggles to maintain sharp object boundaries, which is particularly detrimental for small or intricately shaped objects. The inclusion of this module substantially strengthens the synergy between global and local feature representations.

These results underscore the comprehensive contributions of the CMF and EED modules to the performance of EGFF-Net. The compensatory mechanisms in CMF enable effective cross-modal fusion by leveraging the complementary strengths of RGB and thermal images, while EED refines the salient regions through precise boundary guidance. Together, these modules form a cohesive framework that excels in both accuracy and robustness across diverse RGB-T SOD scenarios.

### 4.5 Analysis for network complexity

To evaluate the computational efficiency and network complexity of our proposed method, we compare it with several state-of-the-art (SOTA) methods in terms of floating point operations (FLOPs) and parameter count, as shown in Table 3. FLOPs, measured in billions (G), quantify the computational effort required for processing each input sample, while the parameter count, measured in millions (M), indicates the model's storage and memory requirements.

**Table 3:** Comparison of network complexity for different methods in RGB-T SOD.

**Dataset**	**MIDD**	**HAI**	**Swin**	**DCL**	**CAVER**	**Wave**	**Ours**
FLOPs(G)	216.72	181.4	124.3	207.31	137.68	26.67	76.32
Params(M)	52.43	59.82	198.7	91.88	55.79	30.17	42.24

Our EGFF-Net achieves significantly lower FLOPs than most methods, including MIDD, HAI, and DCL, demonstrating its computational efficiency. Compared to SwinNet, which leverages transformer-based architectures for hierarchical feature fusion, our method reduces computational cost by 38.6%, making it more suitable for real-time applications. Similarly, compared to CAVER, which emphasizes global feature alignment, our EGFF-Net exhibits a 44.6% reduction in FLOPs. While WaveNet achieves the lowest computational cost due to its lightweight wavelet-based MLP architecture, it sacrifices boundary refinement and modality fusion precision, resulting in suboptimal performance in complex scenarios.

In terms of parameter count, EGFF-Net maintains a competitive balance between model size and performance. With only 42.24M parameters, our method achieves a smaller network size than most SOTA methods, such as SwinNet and DCL, while still outperforming them in salient object detection tasks. Although WaveNet employs an efficient wavelet-based MLP and knowledge distillation to reduce computational complexity, its simplified backbone and reliance on lightweight feature extraction can lead to challenges in capturing intricate boundary details and complex cross-modal interactions, particularly in cluttered or highly dynamic scenes.

The combination of efficient computation and moderate network size ensures that EGFF-Net strikes a compelling balance between real-time performance and robust saliency detection. This makes it well-suited for challenging scenarios with complex backgrounds, weak thermal signals, or misaligned modalities, highlighting its practicality and innovation in the RGB-T SOD domain.

## 5 Conclusion

We proposed a complementary multimodal information fusion technique for RGB-T salient object detection (SOD). Specifically, our approach introduces a cross-modal feature extraction module, which captures saliency information from both RGB and thermal images, leveraging the complementary properties of the two modalities. This module enhances the salient features of one modality while suppressing background noise, resulting in more robust saliency detection. Furthermore, our edge-guided feature fusion module strengthens the edges of salient regions by utilizing edge information, ensuring sharper object boundaries and improving the overall saliency map.

Our method offers several key advantages compared to prior approaches. First, the cross-modal fusion in our model is more effective than basic concatenation methods typically used in other works, as it extracts both shared and unique information from the RGB and thermal modalities. This enhanced fusion allows for better saliency detection, especially in challenging environments such as cluttered backgrounds or low-light conditions. Additionally, by introducing an edge-guided fusion module, we are able to refine object boundaries, an aspect often overlooked by other models, which leads to more accurate localization of salient objects. Furthermore, our approach is particularly effective at suppressing background noise across modalities, contributing to clearer and more distinct saliency maps.

Despite these strengths, our method has certain limitations. First, the computational complexity of our approach is relatively high due to the added modules for edge-guided fusion and cross-modal interaction, which could hinder its deployment in real-time or resource-constrained applications. Second, while our edge-guided fusion module enhances object boundaries, its effectiveness may diminish in scenarios with highly ambiguous edges or extreme occlusion. Additionally, our current method is designed for image-level RGB-T SOD and has not yet been extended to video-based scenarios, which present additional challenges such as temporal consistency.

Future work could focus on addressing these limitations. Optimizing the computational efficiency of our model, for instance, by leveraging lightweight architectures or hardware-aware design, could make it more suitable for real-time applications. Exploring advanced adaptive fusion mechanisms that dynamically weigh RGB and thermal contributions based on scene complexity could further improve detection accuracy. Moreover, expanding the method to handle video-based RGB-T SOD or other multimodal domains, such as medical imaging or autonomous driving, could unlock new opportunities for multimodal information fusion techniques. Finally, conducting ablation studies on different edge-guided strategies and incorporating temporal dynamics into the model could provide deeper insights into its potential and limitations.

In summary, our experiments on public datasets demonstrate that EGFF-Net achieves state-of-the-art performance in RGB-T SOD, particularly excelling in suppressing background noise and refining object boundaries. By addressing the aforementioned challenges, EGFF-Net can be further developed into a more versatile and efficient solution for multimodal salient object detection.

## Data Availability

Publicly available datasets were analyzed in this study. This data can be found here: https://github.com/mmic-lcl/Datasets-and-benchmark-code.
